# Genomic and transcriptomic analysis of camptothecin producing novel fungal endophyte: *Alternaria burnsii* NCIM 1409

**DOI:** 10.1038/s41598-023-41738-6

**Published:** 2023-09-05

**Authors:** Shakunthala Natarajan, Boas Pucker, Smita Srivastava

**Affiliations:** 1 Plant Biotechnology and Bioinformatics, Institute of Plant Biology and Braunschweig Integrated Centre of Systems Biology (BRICS), TU Braunschweig, 38106 Brunswick, Germany; 2 Department of Biotechnology, Bhupat and Jyoti Mehta School of Biosciences, Indian Institute of Technology Madras, Chennai, 600 036 India

**Keywords:** Genomics, Next-generation sequencing, Sequence annotation, Biotechnology, Computational biology and bioinformatics, Drug development, Comparative genomics, Fungal genomics

## Abstract

Camptothecin is an important anticancer alkaloid produced by particular plant species. No suitable synthetic route has been established for camptothecin production yet, imposing a stress on plant-based production systems. Endophytes associated with these camptothecin-producing plants have been reported to also produce camptothecin and other high-value phytochemicals. A previous study identified a fungal endophyte *Alternaria burnsii* NCIM 1409, isolated from *Nothapodytes nimmoniana,* to be a sustainable producer of camptothecin. Our study provides key insights on camptothecin biosynthesis in this recently discovered endophyte. The whole genome sequence of *A. burnsii* NCIM 1409 was assembled and screened for biosynthetic gene clusters. Comparative studies with related fungi supported the identification of candidate genes involved in camptothecin synthesis and also helped to understand some aspects of the endophyte’s defense against the toxic effects of camptothecin. No evidence for horizontal gene transfer of the camptothecin biosynthetic genes from the host plant to the endophyte was detected suggesting an independent evolution of the camptothecin biosynthesis in this fungus.

## Introduction

**Figure 1:**
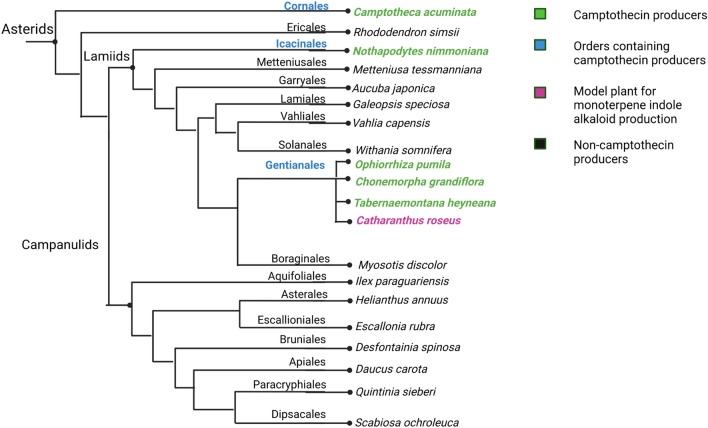
Phylogenetic tree of major camptothecin producing plants and some closely related species. Camptothecin-producing plant species are highlighted in green.

Humans are dependent on plants for a wide variety of natural products. Plants produce the active molecules for a majority of the drugs available in the market^1^. Anticancer drugs derived from plants occupy the top ladder in this list with active compounds like taxol and camptothecin leading in the front^2^. Camptothecin is the third most in-demand alkaloid mainly produced by the medicinal plants, *Camptotheca acuminata* and *Nothapodytes nimmoniana*^3^ (Fig. 1).

Camptothecin binds to the DNA topoisomerase-I, stalls the replication process, and causes cell death. This pharmacological effect makes camptothecin a valuable anticancer molecule. Camptothecin acts only during the S-phase of the cell cycle. Since cancer cells spend a greater proportion of time in S-phase compared to non-cancerous cells, the probability of camptothecin binding to cancer cells is greater^4^. The various drugs derived from camptothecin like topotecan and irinotecan are included in the World Health Organization’s model list of essential medicines^5^. The increasing demand for camptothecin has led to an irrational exploitation of the producing plant species, pushing them towards extinction^6^. Slow growth of these plants makes it difficult to achieve a balance of utilization and regeneration by planting activities. Highly variable amounts of camptothecin in different plants pose another issue that makes it difficult to standardize and produce plant-based extracts with consistent levels of active ingredient^7^. There is also a high degree of heterogeneity in the metabolites produced by the same plant species located in different geographical regions^8^. Moreover, chemical synthesis of products synthesized by plants is difficult and often not eco-friendly due to the use of toxic solvents and harsh reagents^9^. Complex chemical structures like quinolone rings, which are a part of the phytochemical molecules, are extremely difficult to synthesize artificially, making chemical synthesis of such high value phytochemicals unfeasible. Plant cell cultures could be harnessed for the production of these molecules. But such methods are not cost-effective and time consuming when compared to microbial production platforms^10^. The challenges associated with plant-based extraction, culturing and chemical synthesis routes, make microbial synthesis of phytochemicals a suitable method for achieving the goal of sustainable production of these high value products^3, 11^. Plants and their resident microbes called endophytes have established intriguing partnerships through co-evolution. Endophytes are microbes (bacteria or fungi) that reside within the tissues of a plant without harming the host plant^7^. Some of these endophytes are able to synthesize the specialized metabolites produced by the host plants. The endophytes may have gained this ability to produce metabolites from the host plant through horizontal gene transfer (HGT). However, evolutionary and environmental constraints placed on the endophyte for mutual co-existence along with the host, could also cause an independent evolution of the capacity to synthesize the host-produced metabolites^12^. Tolerance towards host-produced metabolites is necessary for the endophyte to ensure a mutual coexistence. This tolerance might be a prerequisite for the evolution of the corresponding biosynthesis pathway. For example, a tolerance for camptothecin might have evolved first in the endophyte which paved the way for a later evolution of the camptothecin biosynthesis pathway^13^.

It is important to delineate the roles of both partners (plant, endophyte) in the secondary metabolite production: (A) the plant and the endophyte could be equal contributors in the metabolite production i.e., plant and endophyte would catalyze complementary reactions and rely on each other for a complete biosynthesis pathway (B) the metabolite production could take place independently in the plant as well as the endophyte. If it is the latter scenario, a sustainable microbial production route for the metabolite could be feasible^12^. Moreover, such a microbe must also be a sustainable producer of the metabolite under industrial conditions to become a suitable production host. Unfortunately, attenuation of the product is frequently observed in the endophyte during subsequent sub-culture cycles^14, 15^. A probable reason that has been proposed is the absence of inducing or silencing genes in the axenic cultures of the endophyte^14^. In summary, microbial production of plant specialized metabolites in an endophyte requires the microorganism to produce the molecule independently of the plant and to show its sustained production over many subcultures.

A number of endophytes were isolated from *N. nimmoniana* in a study by Mohinudeen et al.^3^ Product yield attenuation has been commonly reported among endophytes, placing an element of skepticism on the presence of a host-independent biosynthetic machinery in the endophyte. In contrast to other isolated endophytes, *Alternaria burnsii* NCIM 1409 turned out to be a sustainable producer of camptothecin^11^ and did not exhibit a decline in yield over subsequent sub-cultures (Table 1). The identity of the camptothecin molecules produced by *A. burnsii* NCIM 1409 was supported by tandem mass spectroscopy results^11^. Camptothecin yield was quantified using high performance liquid chromatography^3, 11^. Additional support for the ability of *A. burnsii* NCIM 1409 to endogenously produce camptothecin comes from a study that used ^13^C-labelled glucose feeding in the axenic state^11^. In the light of all these results obtained through various qualitative and quantitative analyses, it can be assumed that *A. burnsii* NCIM 1409 is a *bona fide* producer of camptothecin and has a huge potential to be harnessed as an industrial production source of camptothecin.

**Table 1:** Camptothecin-producing fungal endophytes from two major camptothecin-producing plant species.

Fungal endophytes producing camptothecin	Host plant	Degree of attenuation of camptothecin	References
*Fusarium solani*	*Camptotheca acuminata*	Gradual attenuation observed from 1st to 7th subculture	^15^
*Trichoderma atroviride* LY357	*Camptotheca acuminata*	Stable up to 8 subculture cycles	^16^
*Diaporthe sp.* F18	*Nothapodytes nimmoniana*	Stable up to 6 subculture cycles	^17^
*Alternaria burnsii* NCIM 1409*	*Nothapodytes nimmoniana*	Stable even after 12 continuous subculture cycles	^3, 11^

The * indicates that *Alternaria burnsii* NCIM 1409 is a sustainable producer of camptothecin and does not exhibit yield degeneration like other fungal endophytes. It also signifies that, for the same reason, *Alternaria burnsii* NCIM 1409 has been the focus organism for the present study.

In this study, we obtained important details about the aspects of camptothecin biosynthesis in the endophyte *A. burnsii* NCIM 1409 through genomic analysis. Biosynthetic gene cluster mining and comparative studies with related fungi and host plants revealed insights into the ability of *A. burnsii* NCIM 1409 to produce camptothecin as well as defend itself against it.

## Materials and methods

### Fungal culture, genomic DNA extraction and quality check

The spores of *A. burnsii* NCIM 1409 were inoculated in potato dextrose broth (Himedia) and cultured at 28 °C, 120 rpm for 8 days. Eight-day old fungal liquid suspension was harvested by centrifugation at 10,000 rpm, 4 °C for 15 min. The separated fungal mycelia was ground into powder using liquid nitrogen. The fungal genomic DNA was isolated using the potassium acetate DNA extraction protocol^18^. The isolated DNA was checked for its quality using agarose gel electrophoresis and quantified by NanoDrop measurements. The DNA sample was sent to Eurofins Genomics India Pvt. Ltd. for sequencing.

### Library preparation and quality check for whole genome sequencing

The paired-end sequencing library was prepared from the QC passed genomic DNA sample using Illumina TruSeq Nano DNA Library Prep Kit. Briefly, approximately 200 ng of DNA was fragmented by Covaris M220 to generate a mean fragment distribution of 350 bp. Covaris shearing generates dsDNA fragments with 3' or 5' overhangs. The fragments were then subjected to end-repair. This process converts the overhangs resulting from fragmentation into blunt ends using End Repair Mix. The 3′–5′ exonuclease activity of this mix removes the 3′ overhangs and the 5′–3′ polymerase activity fills in the 5′ overhangs followed by adapter ligation to the fragments. This strategy ensures a low rate of chimera (concatenated template) formation. The ligated products were size selected using AMPure XP beads. The size-selected products were PCR amplified with the index primer as described in the kit protocol indexing adapters were ligated to the ends of the DNA fragments, preparing them for hybridization onto a flow cell.

After obtaining the Qubit concentration for the library and the mean peak size from Agilent TapeStation profile, the PE Illumina library was loaded onto NextSeq500 for cluster generation and sequencing using 2 × 150 bp chemistry. Paired-end sequencing allows the template fragments to be sequenced in both the forward and reverse directions on NextSeq500. The adapters were designed to allow selective cleavage of the forward strands after re-synthesis of the reverse strand during sequencing. The copied reverse strand was then used to sequence from the opposite end of the fragment.

### Genome sequence generation, and assembly

The genome of *A. burnsii* NCIM 1409 was sequenced on an Illumina NextSeq500 at Eurofins Genomics India Pvt. Ltd. FASTQ files obtained through sequencing were quality checked using FastQC (v-0.11.9)^19^ and low quality reads were trimmed using Trimmomatic (v-0.39)^20^ (see Supplementary Methods for details). The genome assembly of the endophyte was generated using SPAdes (v-3.15.5)^21^ (see Supplementary Methods for details). The assembly quality was checked using QUAST (v-5.2.0)^22^ (see Supplementary Methods for details) and assembly statistics were obtained using a custom python script (contig_stats.py^23^).

### Fungal culture for RNA isolation

The spores of *A. burnsii* NCIM 1409 were inoculated in potato dextrose broth (Himedia) and cultured at 28 °C, 120 rpm for 8 days. One day old fungal culture was harvested by centrifugation at 13,500×*g*, 4 °C for 10 min, washed in phosphate-buffered saline. Three one-day old fungal samples were harvested thus followed by snap-freezing using liquid nitrogen, sealing and storage at − 80 °C. Similarly, eight day old fungal culture was harvested by centrifugation at 13,500×*g*, 4 °C, for 10 min, washed in phosphate-buffered saline. Three eight-day old fungal samples were harvested thus followed by snap-freezing using liquid nitrogen, sealing and storage at − 80 °C. All the six samples (three replicates on day 1 and three replicates on day 8) were dispatched on dry ice to Eurofins Genomics India Pvt. Ltd. for RNA isolation and RNA-seq.

### RNA isolation and quality check

Total RNA was isolated from the received fungal pellet using conventional TRIzol method followed by column purification using Quick RNA Plant MiniPrep Kit (Zymo Research). The qualities and quantities of the isolated RNA were checked on NanoDrop followed by Agilent TapeStation using High Sensitivity RNA ScreenTape.

### RNA-seq library preparation and quality check

The RNA-Seq paired end sequencing libraries were prepared from the QC passed RNA samples using NEBNext^®^ Ultra™ II Directional RNA Library Prep Kit for Illumina (NEB) as per manufacturer’s instruction. Briefly, mRNA was enriched from the total RNA using Poly-T attached magnetic beads, followed by enzymatic fragmentation, first strand cDNA conversion using NEBNext First Strand Synthesis Enzyme Mix to facilitate RNA dependent synthesis. The 1st strand cDNA served as a template to synthesize the second strand using the second strand mix. The dscDNA was then purified using AMPure XP beads followed by A-tailing, adapter ligation and then enriched by limited no of PCR cycles.

### Transcriptome analysis with RNA-seq

After obtaining the Qubit concentration for the libraries and the mean peak sizes from Agilent TapeStation profile, the PE Illumina libraries were loaded onto NovaSeq6000 for cluster generation and sequencing. Paired-end sequencing allows the template fragments to be sequenced in both the forward and reverse directions on NovaSeq6000. The adapters were designed to allow selective cleavage of the forward strands after re-synthesis of the reverse strand during sequencing. The copied reverse strand was used to sequence from the opposite end of the fragment.

### Genome annotation

The RNA-seq reads of the endophyte were used as reference for the structural annotation process. Protein hints from a closely related *Alternaria* strain^24–26^ were also integrated with the RNA-Seq hints. BRAKER2^27^ and TSEBRA (v-1.0.3)^28^ (see Supplementary Methods for details) were used to produce the structural genome sequence annotation. The completeness of the resulting annotation was assessed using BUSCO v5.4.2^29^ (see Supplementary Methods for details). The lineage dataset used for BUSCO assessment was pleosporales_odb10^29^, as *Alternaria* fungi belong to the *Pleosporales* order. The CDS and peptide FASTA files were obtained from the genomic FASTA and GFF3 file using a custom python script (get_peps_from_gff3.py). The functional annotation of the predicted genes was obtained using InterProScan5^30^ (Supplementary Table S1) (see Supplementary Methods for details on parameters used). The gene prediction was cleaned to adhere to ENA specifications for data submission. During that process, two genes with duplicated feature location identifiers were removed and sequences shorter than 100 bp were removed.

### Identification of biosynthetic gene clusters in the endophyte

In fungi, specialized metabolism genes encoding proteins that participate in the same biosynthetic pathway are often genomically clustered. Based on the possibility of genes involved in camptothecin synthesis being clustered, the specialized metabolite gene clusters in the fungal endophyte were predicted via antiSMASH 6.1.1^31^ (see Supplementary Methods for details).

### Investigation of horizontal gene transfer (HGT) between endophyte and host plant(s)

**Figure 2:**
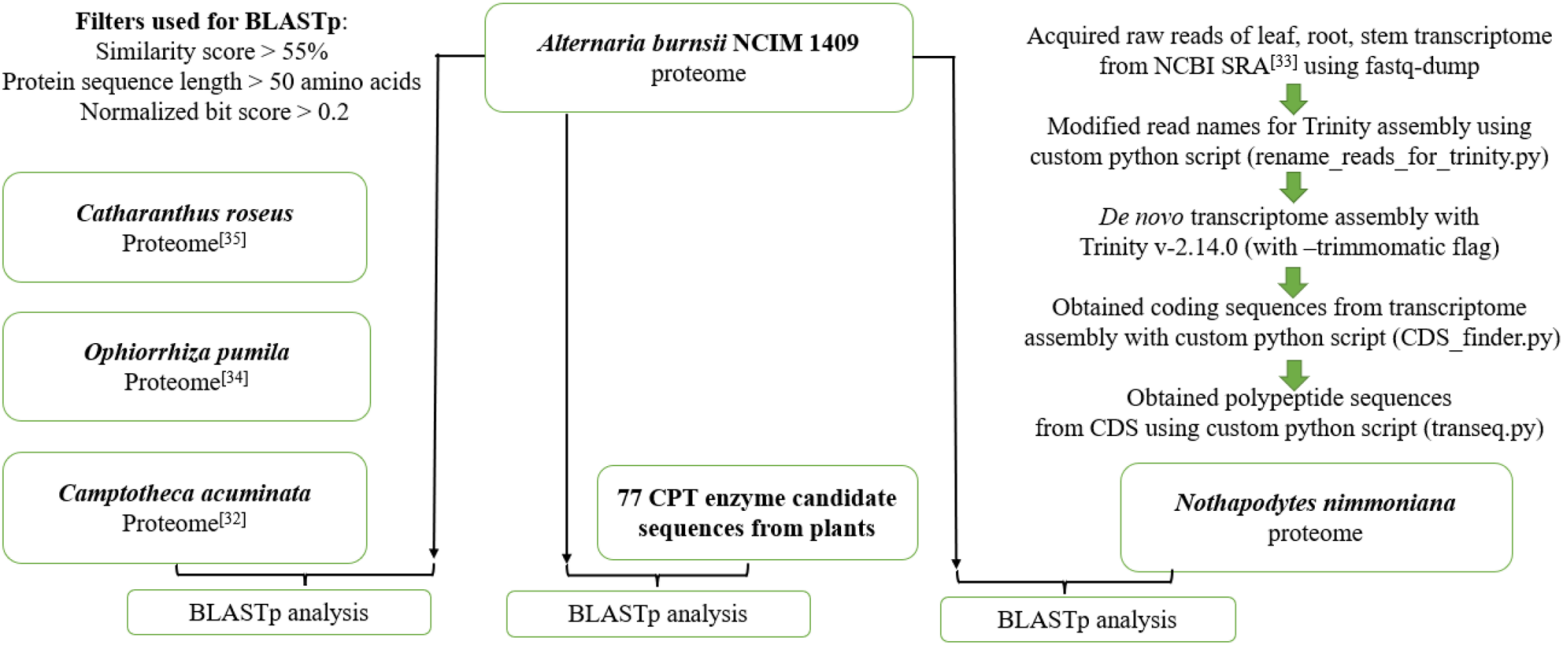
Schematic representation of horizontal gene transfer investigation.

Peptide sequences of three camptothecin producing plants—*C. acuminata*^32^, *N. nimmoniana*^33^, and *O. pumila*^34^, and peptide sequences of a monoterpene indole alkaloid producing plant—*C. roseus*^35^, were subjected to a BLASTp analysis against the endophyte’s peptide sequences to investigate the occurrence of horizontal gene transfer from the plants to the endophyte. Additionally, peptide sequences of key enzymes involved in camptothecin and MIA synthesis in these plants, were also separately retrieved from the NCBI protein database and compared against the *A. burnsii* peptide sequences using BLASTp to avoid missing potential hits indicating horizontal gene transfer (Fig. 2). Sequences of common enzymes involved in the mevalonate pathway like acetyl-CoA acetyltransferase were not found in the CPT producing plants’ taxonomy or MIA producing plants’ taxonomy in the NCBI repository (Supplementary Table S2). Hence, few such enzyme candidates belonging to the mevalonate pathway common to most plants were taken from related plants like *W. somnifera* belonging to the *Asterids* clade, same as the MIA and CPT producing plants. The BLASTp results (Supplementary Table S3) were processed using a custom python scripts (blast2best.py, process_blast_results_v2.py) that applied the following filters to select a gene from the endophyte as a potential candidate, (1) the protein sequence must be longer than 50 amino acids and (2) show a similarity of greater than 55% to the target plant sequence, and (3) have a normalized bit score greater than 0.2. The normalized bit score is calculated by dividing the bit score of a hit against a hit of the query sequence against itself.

### Comparative genomics studies with related fungal organisms

Comparative studies of *A. burnsii* NCIM 1409 against closely related *Alternaria* fungi and other specialized metabolite producing fungi (Supplementary Table S4) were carried out using OrthoFinder2^36^. This analysis helped identify the genes unique to *A. burnsii* NCIM 1409 and lacking in the other fungi, by parsing the OrthoFinder2 results (Supplementary Table S5) using a custom python script (Process_orthofinder_for_unique_genes.py). Since gene duplications are important drivers of evolution and help organisms to acquire new functions^37^, it was speculated that the gene duplicates in the endophyte could reveal candidates involved in camptothecin production. Synteny analysis with JCVI/MCscan^38^ using a custom python script (jcvi_pairwise_synteny.py) was used to obtain pairwise syntenic blocks files connecting the annotations of *A. burnsii* NCIM 1409 and the *Alternaria* fungi. The blocks files were processed using a custom python script (Gene_duplications_synteny_v3.py) to obtain gene duplications in the fungal endophyte. Thus the comparative analyses with fungi helped consolidate some camptothecin candidate genes in *A. burnsii* NCIM 1409.

### Investigation of defense mechanism in the endophyte against the toxic effects of camptothecin

To understand the defense mechanism against camptothecin, the DNA topoisomerase I protein sequence of *A. burnsii* NCIM 1409 was retrieved and aligned with the DNA topoisomerase I sequences of *Homo sapiens*, other CPT-producing plants, non-CPT-producing fungi, and a CPT-producing fungus, using MAFFT (v-7.511)^39^. The aligned sequences were examined for specific CPT-resistance conferring mutations with the DNA topoisomerase I sequence from *Homo sapiens* serving as the reference for the amino acid residue positions.

## Results

### Assembly and annotation

The genome assembly of the endophyte comprises 104 contigs, with a total size of 33.2 Mb (Table 2). The N50 value of 832,062 bp indicates high assembly continuity.

**Table 2:** Statistics of the *A. burnsii* NCIM 1409 genome assembly.

Parameters	Values
N50	832,062 bp
Maximal contig length	3,333,410 bp
Minimal contig length	502 bp
Average contig length	319,594 bp
Number of contigs	104
Assembly size	33.2 Mb

The structural annotation of the endophyte harbors 13,351 protein-encoding genes with an average gene size of 1434 bp. The longest gene has a size of 29,413 bp. The BUSCO completeness score of this structural annotation was 98.6% (C: 98.6% [S: 92.1%, D: 6.5%], F: 0.3%, M: 1.1%, n: 6641).

Mevalonate and shikimate pathways eventually lead to camptothecin synthesis and hence enzymes in these pathways are important for camptothecin biosynthesis^40^. The functional annotation of all predicted genes in *A. burnsii* NCIM 1409 identified 37 candidate genes (Supplementary Table S6) in the endophyte that coded for some enzymes in the mevalonate and shikimate pathways (Supplementary Figure).

### Biosynthetic gene clusters in *Alternaria burnsii* NCIM 1409

antiSMASH predicted 25 gene clusters in the fungus. The cluster details and the similarity of some clusters to known clusters are given in Table 3 shown below.

**Table 3:** List of biosynthetic gene clusters predicted in the *A. burnsii* NCIM 1409 using antiSMASH 6.1.1

Cluster	Genes in each cluster	Type	Similarity to known clusters
Cluster 1	g3165-g3173	Terpene	
Cluster 2	g3944-g3958	T1PKS	
Cluster 3	g5907-g5926	TIPKS, NRPS	
Cluster 4	g8313-g8329	NRPS-like	
Cluster 5	g9283-g9298	NRPS-like	
Cluster 6	g9546-g9566	NRPS-like	
Cluster 7	g10734-g10752	T1PKS	
Cluster 8	g11032-g11040	Terpene	
Cluster 9	g11572-g11587	NRPS	Dimethylcoprogen—NRP; 100% similarity
Cluster 10	g11705-g11721	NRPS	
Cluster 11	g12-g25	NRPS-like, Indole	
Cluster 12	g1114-g1122	Terpene	
Cluster 13	g1372-g1388	T1PKS	Alternapyrone—Polyketide; 100% similarity
Cluster 14	g2119-g2127	Terpene	
Cluster 15	g5532-g5540	Terpene	PR-toxin—Terpene; 50% similarity
Cluster 16	g6652-g6667	NRPS-like	
Cluster 17	g7285-g7299	T1PKS	Abscisic acid—Polyketide; 25% similarity
Cluster 18	g7433-g7443	T1PKS	Melanin—Polyketide; 100% similarity
Cluster 19	g7661-g7674	T1PKS	Depudecin—Polyketide iterative type I; 33% similarity
Cluster 20	g7683-g7689	Terpene	Squalestatin S1—Terpene; 40% similarity
Cluster 21	g7842-g7856	NRPS-like	
Cluster 22	g8594-g8603	NRPS-like	
Cluster 23	g9129-g9140	T1PKS	
Cluster 24	g9932-g9947	T1PKS	Betaenone A/ betaenone B/ betaenone C—Polyketide; 62% similarity
Cluster 25	g10060-g10068	NRPS-like	

### Comparison of the *A. burnsii* NCIM 1409 gene set against genes of host plants

Endophytes reside in the host plant and acquire the ability to produce secondary metabolites. Hence, horizontal gene transfer of biosynthetic genes between them could be a possible occurrence^12, 15, 41^. If horizontal gene transfer of genes is detected, then this could reveal more genes involved in camptothecin synthesis in *A. burnsii* NCIM 1409 with a greater reliability. That would in turn provide a deeper understanding of camptothecin production in the endophyte. Camptothecin production has been found to be reported in 43 plant species belonging to different orders^42^. For example, *C. acuminata* belonging to *Cornales*, *N. nimmoniana* belonging to *Icacinales*, and *Ophiorrhiza pumila* belonging to *Gentianales*, are all producers of camptothecin^43^. This phylogenetically scattered occurrence of camptothecin production capacity across different plant species is an intriguing phenomenon. Another medicinal plant called *Catharanthus roseus*, despite being a non-producer of camptothecin, is widely regarded as the model plant for monoterpene indole alkaloid synthesis and shares the same enzymes found in camptothecin producers, till a certain stage in its pathway leading to a wide variety of MIAs like vinblastine^35, 43^. It is observed through comparative analyses of these medicinal plants that, the enzymes catalyzing the upper parts of the pathway leading to camptothecin or other MIAs in case of *C. roseus* share a high degree of similarity, whereas, towards the downstream steps of the pathway, the enzyme similarity, even among camptothecin producers becomes very low^43^. Due to such a divergence exhibited in the pathways leading to camptothecin and other MIAs in plants, the peptide sequences of all the four aforementioned plants were included in this investigation for horizontal gene transfer occurrence.

Despite extensive searches, no evidence was detected for horizontal gene transfer of the camptothecin biosynthesis from the host plant to the endophyte. A BLASTp screen of peptide sequences of *C. acuminata* and *C. roseus* against the endophyte revealed no significant hits. The BLASTp of peptide sequences of *N. nimmoniana* and *O. pumila* against the endophyte identified one gene (g616.t1) that showed a similarity greater than 55% and 59%, respectively, and a good normalized bit score. The BLASTp of candidates retrieved from the National Center for Biotechnology Information (NCBI) against that of the endophyte also identified g616.t1 as the best candidate. A similarity of 60% was observed in a comparison against the acetyl-CoA acetyltransferase sequence from *W. somnifera.* The functional annotation of this particular gene in the endophyte also indicated a function as acetyl-CoA acetyltransferase. This gene encodes for the initial enzyme in the mevalonate pathway, found in most plants and fungi. Also, the similarity values of around 60% do not indicate an evolutionarily recent HGT event. Further, those endophyte sequences that show high similarity to the plant sequences are involved in core functions like central metabolism, transcription, translation, which are highly conserved across organisms (Supplementary Table S7). Hence, there was no evidence found to suggest horizontal gene transfer from the host plant to the endophyte.

### Comparison of the *A. burnsii* NCIM 1409 gene set against related fungi

Orthogroups were identified between a selection of fungal species to identify shared and private genes. The OrthoFinder2 analysis revealed 26 genes in the *A. burnsii* NCIM 1409 that were present in orthogroups not shared with genes from other investigated fungi. There were also some genes in the endophyte that were not present in the orthogroups file. There were also some species-specific single copy genes that could be unique to *A. burnsii* NCIM 1409, that were found by processing the OrthoFinder2 results using a custom python script (Process_orthofinder_for_unique_genes.py). In total, 233 unique genes were identified in the endophyte that did not have orthologs in other fungi. Since the comparison also included another fungus *Xylaria* sp. M71 that produces a camptothecin derivative (10-hydroxycamptothecin)^44^, genes that were only shared between *A. burnsii* NCIM 1409 and X. sp M71 were also searched. There was only one gene (g3550.t1) that was present only in these two organisms and it encoded a SAM-dependent methyltransferase. Hence, it was also included as a camptothecin candidate gene in *A. burnsii* NCIM 1409 along with the other genes mined from the OrthoFinder2 analysis.

Next, the search for gene duplicates based on synteny analysis yielded 215 gene duplicates in *A. burnsii*, while all the other *Alternaria* fungi showed only one corresponding gene. Some of these candidate genes were identified by both the OrthoFinder and synteny analyses, making them more important ones for further investigation (Supplementary Table S6). After obtaining the peptide sequences of the identified candidates, those sequences that were too short (less than 30 amino acids in length) and those that appeared to be artifacts were removed from the candidate gene list. Thus, after cleaning the candidate genes using the above-mentioned criteria, the comparative analyses with other fungi helped obtain a total of 449 CPT candidate genes in the endophyte (Supplementary Table S6, Supplementary Results).

### Analysis of DNA topoisomerase I in *Alternaria burnsii* NCIM 1409

Since camptothecin is a toxic molecule that inhibits replication by binding to DNA topoisomerase I, it is quite interesting to see how camptothecin producers avoid detrimental impacts of their end product. Knowledge about this defense mechanism is important to understand camptothecin biosynthesis in the endophyte. A multiple sequence alignment (MSA) showed various critical amino acid residues of DNA topoisomerase I sequence of *A. burnsii* NCIM 1409. It also helped look out for well-known mutations modulating camptothecin and DNA topoisomerase binding, in the DNA topoisomerase I sequence of *A. burnsii* NCIM 1409 (Supplementary Tables S8−S12).

The camptothecin-producing endophytes, as well as non-camptothecin producing close relatives and distant fungi did not show the three camptothecin-resistance conferring mutations—N421K, L530I, N722S found in camptothecin producing plants^45^. Sequences of all species displayed N in the position of interest, which matches the amino acids in the corresponding sequences of camptothecin-producing plant *O. pumila* and non-camptothecin plant *C. roseus.* All the fungi included in this analysis have L corresponding to L530 and share this with camptothecin-producing *C. acuminata,* and non-camptothecin plant *C. roseus.* All of them have N corresponding to N722 and share this with N in non-camptothecin plant *C. roseus* and camptothecin producer *N. nimmoniana.* The catalytic amino acid residues and some camptothecin resistance conferring wild-type amino acid residues are highly conserved in camptothecin producers and non-camptothecin producers across all organisms. For example, N352 and F361, two residues in the binding region, are important for camptothecin resistance^46^. They are found in all the sequences used in this study and are not exclusive to camptothecin producers. Among other camptothecin resistance conferring mutations in the binding region, only M370T^47^ mutation is observed in the fungal sequences (except *N. aurantialba*) and in *N. nimmoniana.* Again, this mutation is not exclusive to camptothecin-producing endophytes. The other camptothecin resistance conferring mutations were not detected in the binding regions in the sequences considered here. The remaining residues in the binding region (E356, H367, V502, Y619, D725)^13^ were found to be intact without any variation across all the sequences. Critical residues in DNA topoisomerase I sequences from two fungi—*A. burnsii* NCIM 1409 producing camptothecin, and *Xylaria sp. M71* producing 10-Hydroxy-camptothecin show no variation and are highly similar to each other as well as to other compared sequences of fungi that are not reported producers of camptothecin.

## Discussion

The genomic and transcriptomic analysis of the novel fungal endophyte *A. burnsii* NCIM 1409 was carried out for the first time in this study. The assembled genome sequence provides a basis for future studies. RNA-seq data sets support the predicted gene models. Potential functions have been assigned to most genes.

The specialized metabolite genes in fungi can be under the control of a shared regulatory network resulting in simultaneous activity of all genes in the gene cluster. Among such clusters, terpene biosynthesis gene clusters seem to play a major role in facilitating signal transfer between the host and endophyte during plant–microbe interactions^48^. The 25 gene clusters identified in *A. burnsii* NCIM 1409 could provide potential clues to understand specialized metabolite synthesis in the endophyte. With camptothecin being a monoterpene indole alkaloid, the terpene clusters and the hybrid NRPS-like, indole cluster 11 (Table 2), could harbor potential genes involved in camptothecin biosynthesis. The novel endophyte in focus was isolated from *N. nimmoniana.* The camptothecin biosynthesis pathway in the host plant is complex, starting from the mevalonate (MVA) pathway or the methylerythritol phosphate (MEP) pathway and the shikimate pathway. The MVA or the MEP pathway produce isoprenoid molecules that are modified further to produce secologanin. The shikimate pathway produces tryptophan that condenses with secologanin to produce ‘strictosidine synthase’ (STR)—the parent molecule for a wide variety of MIAs. The steps of the pathway leading to camptothecin, after the Pictet Spengler reaction catalyzed by STR, have eluded researchers so far^33, 49^. Since the endophyte isolated from the host, retained its ability to produce camptothecin for over 12 sub-culture cycles independent of the host^3, 11^, it was expected that during its life cycle in the host tissues, some of the key genes encoding crucial enzymes in camptothecin synthesis might have been transferred from the host plant to the endophyte. This prompted the investigation to assess for the occurrence of horizontal gene transfer between the host and the fungus. If horizontal gene transfer would have been detected, then that could have revealed more genes involved in camptothecin synthesis in *A. burnsii* NCIM 1409. However, the present study does not reveal any evidence for HGT from the host to the endophyte. This observation is also corroborated by a few other studies that investigated HGT from host plants to endophytes producing similar specialized metabolites. Extremely low similarity was found between fungal sequences of taxol producing fungi isolated from the yew tree and taxane specific genes in the yew tree^50^. HGT was also found to be absent between the paclitaxel producing fungus *Penicillium aurantiogriseum* NRRL 62,431 isolated from the hazel plant (*Corylus avellana)*^51^*.* The researchers hypothesize that the taxol-producing genes in the endophyte possess a completely different evolutionary pattern. These reports and the inferences from the present study indicate the possibility for independent evolution of camptothecin biosynthesis in *A. burnsii* NCIM 1409. It also needs to be emphasized here that, while this work does not report any evidence for horizontal gene transfer, at the same time, it does not completely negate the possibility for such an HGT event. Based on the investigations for HGT, this study proposes a plausible possibility for an independent camptothecin biosynthesis machinery in the endophyte. Although the HGT hypothesis was not helpful in finding camptothecin candidate genes in the endophyte, comparative studies with other fungi revealed a number of candidate genes. The fungi included in the comparative analysis were classified into three bins—‘camptothecin producers’, ‘camptothecin non-producers’ and ‘Related product producers’ (fungi producing specialized metabolites like taxol and other specialized metabolites similar to camptothecin, but do not produce camptothecin itself) (Supplementary Table S4). This classification developed for the comparison was used as a basis to identify the candidate genes in the novel endophytes with respect to the other fungi. The endophyte genes that are not shared with the other (all the three classes) fungi can be considered to be specifically unique to itself. Camptothecin synthesis being a unique trait found in this endophyte, these unique genes could possibly hold the key for unraveling more aspects of camptothecin biosynthesis in the fungus and in fungi in general. Fungi could form a reservoir of camptothecin biosynthesis enzymes with properties desirable for biotechnological applications. Genes that are exclusively shared between camptothecin producers and are absent from other fungi, could be promising targets of future studies.

Synteny analysis was crucial for the identification of gene duplications in the endophyte with respect to closely related *Alternaria* fungi. Gene duplications are major drivers of evolutionary innovations as they enable neo- and subfunctionalization^37^. Duplications lower the constraints on natural selection processes, and play a significant role in causing new functions to appear in organisms^52^. Since camptothecin production is a novel trait in the endophyte, such duplications could play an important role in camptothecin synthesis. While focusing on the novel endophyte as a source organism for camptothecin production, it would be important to understand the mechanism being used by the endophyte to protect itself against the toxic effects of camptothecin. This tolerance mechanism could be harnessed to enhance the camptothecin yield from the producing organism. It was reported that the DNA Topoisomerase I in plants possess certain mutations that make them self-resistant to the camptothecin molecule they produce^45^. There have not been many reports investigating the resistance mechanism in camptothecin-producing endophytes, except for one by Kusari and co-workers^13^. However, no specific resistance-endowing mutations were detected in another camptothecin-producing endophyte isolated from *C. acuminata.* It was proposed that the fungus could be using some other mechanism to protect itself from the deleterious effects of camptothecin^13^. Based on the DNA topoisomerase I analysis of the endophyte in this work, it is evident that, camptothecin resistance and camptothecin production in fungal endophytes need not have co-evolved like the co-evolution of camptothecin resistance and camptothecin production in camptothecin-producing plants, as proposed by^45^. This can be further explained by the observation that the camptothecin producing endophyte *A. burnsii* NCIM 1409 and non-camptothecin producing fungi, both have some of the camptothecin resistance conferring amino acid residues. These inferences agree with the conclusions put forth by Kusari and co-workers^13^, which convey that fungi that colonize a toxic metabolite producing plant like a camptothecin producing one, must possess innate resistance to overcome the toxicity, and invade the plant. Of the invaders some may prove to be camptothecin producers while, some may not, as seen in the isolation of several camptothecin producing and non-producing endophytes from different camptothecin producing plants like *C. acuminata*^15^ and *N. nimmoniana*^11^. Although previously studied and expected mutations in DNA topoisomerase I were not present in the fungal sequences, it remains possible that these fungi could have evolved completely different mutations to combat camptothecin as hinted by^13^. But it is interesting to note the fact that *A. burnsii* NCIM 1409 exhibits resistance to camptothecin in a dose dependent manner^3^. This reduces the chances of mutations appearing in the DNA topoisomerase I sequence to create a completely foolproof camptothecin resistant sequence, and this view is also buttressed by dose-dependent resistance to camptothecin exhibited by camptothecin-producing fungus *Phomopsis sp.* isolated from *N. nimmoniana*^53^. This could mean that *A. burnsii* NCIM 1409 could have developed entirely new ways of resisting camptothecin apart from variation in its DNA topoisomerase I sequence. Or rather than resisting camptothecin, we speculate that the fungus might tolerate camptothecin up to a particular level, after which the fate of camptothecin within the producer needs to be investigated further.

## Conclusion

Our work provided insights into the camptothecin biosynthesis ability of the fungal endophyte *A. burnsii* NCIM 1409, an camptothecin producer. A lack of evidence for horizontal transfer of camptothecin biosynthesis genes from the host plant to the endophyte is suggestive of an independent evolution of the camptothecin biosynthesis pathway in the fungus. Comparative studies with other fungi narrowed down camptothecin candidate genes in the endophyte that could be validated in further analyses. The fungal endophyte does not possess unique and specific camptothecin-resistance conferring variations in its DNA topoisomerase I sequence. It seems to use a distinct mechanism to protect itself from the deleterious effects of the camptothecin molecule it produces. This work identified candidate genes for camptothecin biosynthesis in the endophyte, which need further analyses to unravel the camptothecin biosynthetic route in the endophyte. Further work needs to focus on an analysis of enzyme activity in the upstream camptothecin pathway, encoded by the identified candidate genes. Next, integration of the whole genome data with transcriptome data from an elaborate time-series based RNA-seq experiment is also crucial in completing the camptothecin quest in the endophyte. To conclude, this study paved the way for further exploration of the genetic mechanisms underlying the camptothecin production in the endophyte. In the future, this could help to establish *A. burnsii* NCIM 1409, as a sustainable production platform for camptothecin on a large scale.

### Supplementary Information

Supplementary Figure 1.

Supplementary Information 2.

Supplementary Information 3.

Supplementary Table S1.

Supplementary Table S2.

Supplementary Table S3.

Supplementary Table S4.

Supplementary Table S5.

Supplementary Table S6.

Supplementary Table S7.

Supplementary Tables.

## Data Availability

The Whole Genome Shotgun project has been deposited at ENA under the accession ERZ18273747. The ENA project accession number is PRJEB61631. The plant and fungal annotations generated as a part of the study can be accessed at: https://github.com/ShakunthalaNatarajan/GenomeAssembly_AburnsiiNCIM1409/tree/main/Annotations. The Multiple sequence alignment FASTA file can be accessed at: https://github.com/ShakunthalaNatarajan/GenomeAssembly_AburnsiiNCIM1409/tree/main/MSA_file.

## Abbreviations

PCR: Polymerase chain reaction
PE: Paired-end
RNA-seq: RNA sequencing
cDNA: Complementary deoxyribonucleic acid
HGT: Horizontal gene transfer
MIA: Monoterpene indole alkaloids
STR: Strictosidine synthase
MSA: Multiple sequence alignment
MVA: Mevalonate pathway
MEP: Methylerythritol pathway
T1PKS: Type I polyketide synthase
NRPS: Non ribosomal peptide synthetase
SAM-dependent methyltransferase: S-adenosyl-methionine-dependent methyltransferase

## Code availability

The scripts and codes used in this study can be accessed at: https://github.com/ShakunthalaNatarajan/GenomeAssembly_AburnsiiNCIM1409.
