# Supplementary material for: Genomic and transcriptomic analysis of camptothecin producing novel fungal endophyte: Alternaria burnsii NCIM 1409
Source: Sci Rep. 2023 Sep 5;13:14614. doi: 10.1038/s41598-023-41738-6 (PMC10480469; doi:10.1038/s41598-023-41738-6)
Supplement: Supplementary file 1 — Supplementary Figure 1. [file 41598_2023_41738_MOESM1_ESM.pdf]

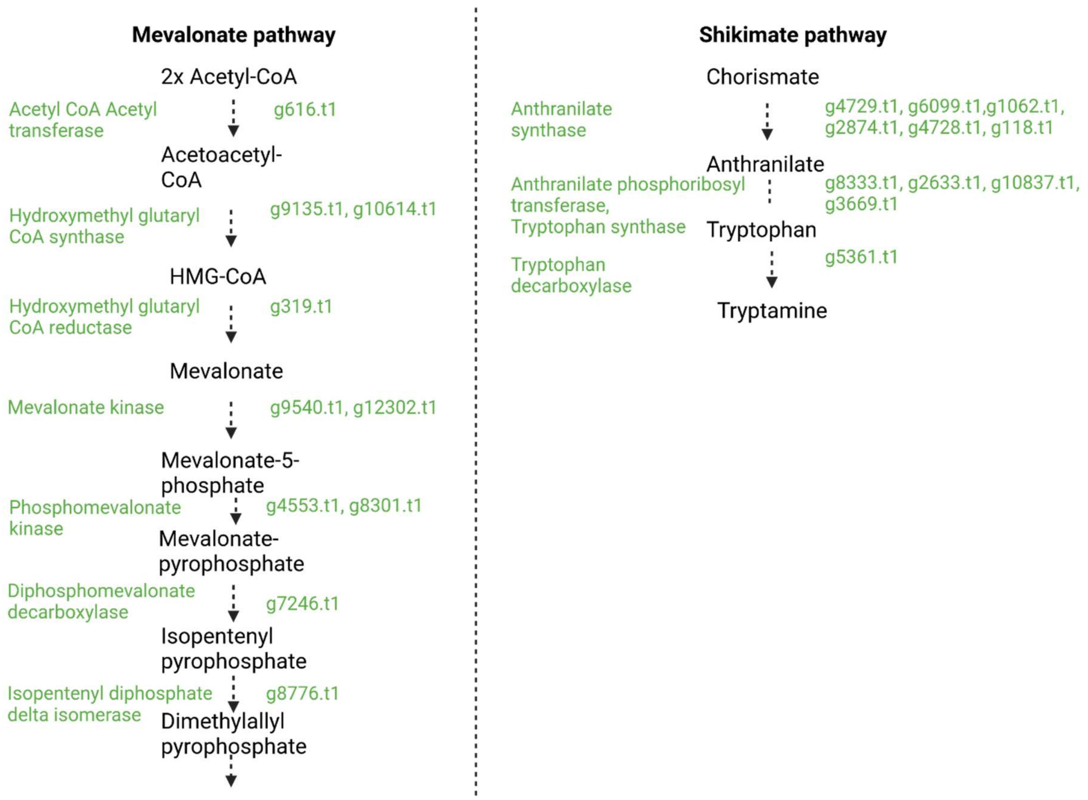

Supplementary Figure: Candidate genes of the endophyte involved in mevalonate and shikimate pathways
