# Supplementary material for: Genomic and transcriptomic analysis of camptothecin producing novel fungal endophyte: Alternaria burnsii NCIM 1409
Source: Sci Rep. 2023 Sep 5;13:14614. doi: 10.1038/s41598-023-41738-6 (PMC10480469; doi:10.1038/s41598-023-41738-6)
Supplement: Supplementary file 11 — Supplementary Tables. [file 41598_2023_41738_MOESM11_ESM.pdf]

Table S8: MSA results to explore the presence of plant CPT-resistance conferring mutations

**CPT Resistance conferring mutations in CPT producing plants →**	**N421K**	**L530I**	**N722S**
Homo sapiens	Q	L	N
Alternaria alternata ATCC11680	N	L	N
Alternaria alternata ATCC66891	N	L	N
Alternaria alternata BMP0270	N	L	N
Alternaria alternata SRC	N	L	N
Alternaria sp. MG1	N	L	N
Alternaria SPS2	N	L	N
Alternaria burnsii NCIM 1409*	N	L	N
Cladosporium cladosporioides MD2 transcript 1	N	L	N
Cladosporium cladosporioides MD2 transcript 2	N	L	-
Cladosporium cladosporioides MD2 transcript 3	N	L	N
Penicillium aurantiogriseum NRRL 62431	N	L	N
Xylaria sp M71 transcript 1*	N	L	N
Xylaria sp M71 transcript 2*	N	L	N
Xylaria sp M71 transcript 3*	N	L	N
Naematelia aurantialba	N	L	N
Catharanthus roseus	N	L	N
Ophiorrhiza pumila	N	I	S
Camptotheca acuminata	K	L	S
Nothapodytes nimmoniana	K	I	N

*** Fungal endophyte producing CPT, its derivative; ** Plants producing CPT, its derivatives

Table S9: MSA results indicating the residues involved in the catalytic function of DNA topoisomerase I

Residues involved in catalytic function →	R488	K532	R590	H632	Y723
Homo sapiens	R	K	R	H	Y
Alternaria alternata ATCC11680	R	K	R	H	Y
Alternaria alternata ATCC66891	R	K	R	H	Y
Alternaria alternata BMP0270	R	K	R	H	Y
Alternaria alternata SRC	R	K	R	H	Y
Alternaria sp. MG1	R	K	R	H	Y
Alternaria SPS2	R	K	R	H	Y
*Alternaria burnsii NCIM 1409****	R	K	R	H	Y
Cladosporium cladosporioides MD2 transcript 1	R	K	R	H	Y
Cladosporium cladosporioides MD2 transcript 2	R	K	R	H	Y
Cladosporium cladosporioides MD2 transcript 3	R	K	R	H	Y
Penicillium aurantiogriseum NRRL 62431	R	K	R	H	Y
*Xylaria sp M71 transcript 1****	R	K	R	H	Y
*Xylaria sp M71 transcript 2****	R	K	R	H	Y
*Xylaria sp M71 transcript 3****	R	K	R	H	Y
Naematelia aurantialba	R	K	R	H	Y
Catharanthus roseus	R	K	R	H	Y
*Ophiorrhiza pumila***	R	K	R	H	Y
*Camptotheca acuminata***	R	K	R	H	Y
*Nothapodytes nimmoniana***	R	K	R	H	Y

*** Fungal endophyte producing CPT, its derivative; ** Plants producing CPT, its derivatives

Table S10: MSA results to investigate the mutations involved in modulating drug binding to DNA topoisomerase I

Mutations involved in drug binding →	N352A	F361S	G363S /V/C	G365S	R364H	M370 T	E418K	I420V	G503S	D533G	A653P	G717V/ F	T729A
Homo sapiens	N	F	G	G	R	M	E	I	G	D	A	G	T
***Alternaria alternata* ATCC11680**	N	F	G	G	R	T	E	I	G	D	G	G	T
***Alternaria alternata* ATCC66891**	N	F	G	G	R	T	E	I	G	D	G	G	T
***Alternaria alternata* BMP0270**	N	F	G	G	R	T	E	I	G	D	G	G	T
***Alternaria alternata* SRC**	N	F	G	G	R	T	E	I	G	D	G	G	T
***Alternaria* sp. MG1**	N	F	G	G	R	T	E	I	G	D	G	G	T
***Alternaria* SPS2**	N	F	G	G	R	T	E	I	G	D	G	G	T
Alternaria burnsii* NCIM 1409**	N	F	G	G	R	T	E	I	G	D	G	G	T
***Cladosporium cladosporioides* MD2 transcript 1**	N	F	G	G	R	T	E	I	G	D	G	G	T
***Cladosporium cladosporioides* MD2 transcript 2**	N	F	G	G	R	T	E	I	G	D	G	G	-
***Cladosporium cladosporioides* MD2 transcript 3**	N	F	G	G	R	T	E	I	G	D	G	G	T
***Penicillium aurantiogriseum* NRRL 62431**	N	F	G	G	R	T	E	I	G	D	G	G	T
Xylaria* sp M71 transcript 1**	N	F	G	G	R	T	E	I	G	D	G	G	T
Xylaria* sp M71 transcript 2**	N	F	G	G	R	T	E	I	G	D	G	G	T
Xylaria* sp M71 transcript 3**	N	F	G	G	R	T	E	I	G	D	G	G	T
Naematelia aurantialba	N	F	G	G	R	K	E	V	G	D	A	T	T
Catharanthus roseus	N	F	G	G	R	M	D	I	G	D	E	G	T
Ophiorrhiza pumila**	N	F	G	G	R	V	D	V	G	D	E	S	T
Camptotheca acuminata**	N	F	G	G	R	M	D	I	G	D	E	G	T
Nothapodytes nimmoniana**	N	F	G	G	R	T	D	I	G	D	E	G	T

*** Fungal endophyte producing CPT, its derivative; ** Plants producing CPT, its derivatives

Table S11: MSA results indicating the residues involved in drug binding to DNA topoisomerase I

Residues involved in drug binding	E356	H367	V502	Y619	D725
Homo sapiens	E	H	V	Y	D
Alternaria alternata ATCC11680	E	H	V	Y	D
Alternaria alternata ATCC66891	E	H	V	Y	D
Alternaria alternata BMP0270	E	H	V	Y	D
Alternaria alternata SRC	E	H	V	Y	D
Alternaria sp. MG1	E	H	V	Y	D
Alternaria SPS2	E	H	V	Y	D
*Alternaria burnsii NCIM 1409****	E	H	V	Y	D
Cladosporium cladosporioides MD2 transcript 1	E	H	V	Y	D
Cladosporium cladosporioides MD2 transcript 2	E	H	V	Y	-
Cladosporium cladosporioides MD2 transcript 3	E	H	V	Y	D
Penicillium aurantiogriseum NRRL 62431	E	H	V	Y	D
*Xylaria sp M71 transcript 1****	E	H	V	Y	D
*Xylaria sp M71 transcript 2****	E	H	V	Y	D
*Xylaria sp M71 transcript 3****	E	H	V	Y	D
Naematelia aurantialba	E	H	V	Y	D
Catharanthus roseus	E	H	V	Y	D
*Ophiorrhiza pumila***	E	H	V	Y	D
*Camptotheca acuminata***	E	H	V	Y	D
*Nothapodytes nimmoniana***	E	H	V	Y	D

*** Fungal endophyte producing CPT, its derivative; ** Plants producing CPT, its derivatives

Table S12: Analysis of important residues and mutations in DNA Topoisomerase I of all organisms used to obtain the MAFFT alignment file

**Mutation/Residue in DNA Top I (Number according to Human DNA Top I)**	**Significance of the residue and (or) its mutation**	**Reference(s)**	**Inference from the current study**
N421K	N to K mutation confers CPT resistance	[45]	Present only in DNA Top I sequences of *C. acuminata* and *N. nimmoniana;* Fungi have N421 like *O. pumila* and *C. roseus*
L530I	L to I mutation confers CPT resistance	[45]	Present only in DNA Top I sequences of *O. pumila* and *N. nimmoniana*. All other sequences show L530
N722S	N to S mutation confers CPT resistance	[45]	Present only in DNA Top I sequences of *C. acuminata* and *O. pumila* with a gap in one of the DNA Top I sequence from *Cladosporium cladosporioides MD2;* All other sequences show N722
R488	Residue involved in catalytic function; contributes to CPT resistance by hampering the water enabled contact of CPT E-ring to itself	[45]	Highly conserved in the DNA Top I sequence from all the organisms included in this study
K532	Residue involved in catalytic function	[13, 45]	Highly conserved in the DNA Top I sequence from all the organisms included in this study
R590	Residue involved in catalytic function	[13, 45]	Highly conserved in the DNA Top I sequence from all the organisms included in this study
H632	Residue involved in catalytic function	[13, 45]	Highly conserved in the DNA Top I sequence from all the organisms included in this study
Y723	Residue involved in catalytic function	[13, 45]	Highly conserved in the DNA Top I sequence from all the organisms included in this study
N352A	N352 shows a dynamic mobile behaviour, plays a role in CPT resistance; N352A mutation	[13, 46]	N352 is present in all the sequences involved. No mutation found.

	renders a CPT sensitive DNA Top I enzyme		
F361S	F361 to S mutation gives a CPT sensitive enzyme	[13, 45]	F361 present in all sequences involved. No mutation found.
G363S/V/C	CPT resistance conferring mutation	[13]	G363 found in all sequences. No mutation
G365S	CPT resistance conferring mutation	[13]	G365 found in all sequences. No mutation
R364H	R364 to H mutation confers resistance to CPT	[13, 46]	R364 present in all sequences involved. No mutation found
M370T	M370T mutation confers CPT resistance in CPT resistance human lung cancer cell lines	[13, 47]	M370T mutation found in all the fungal sequences used, except for that of *N. aurantialba* which had a M370K; *N. nimmoniana* showed the M470T mutation as well. While *O. pumila* had a V, *C. roseus, C. acuminata* had M; M370T mutation is present in other non-CPT producing closely as well as distantly related fungi.
E418K	E418K mutation confers CPT resistance in CPT resistant and part revertant human nasopharyngeal carcinoma cell lines (HONE-1)	[58]	E418 found in human and fungal sequences. E418D mutation found in all plant sequences.
I420V	I420 present in drug binding site of DNA Top I. I to V mutation does not give any altered sensitivity to CPT. But this could be an important residue to look at, as it is present in the drug binding site.	[45]	I420 found in all sequences, except in *N. aurantialba* which has a V420, and *O. pumila* which again has a V420
G503S	Mutation of G503 confers CPT resistance due to the mutation making the E-ring contact of CPT and DNA Top I impossible	[13, 46]	G503 highly conserved across all sequences. No mutation
D533G	D533 is a residue that is needed for enzyme sensitivity to CPT;	[13, 46]	D533 highly conserved across all sequences. No mutation.

	D533G mutation potentially confers CPT resistance		
A653P	A653P mutation increases the rate of enzyme-catalyzed DNA relegation, and thereby confers CPT resistance	[13]	A653 in Human sequence; E653 in CPT producing and non-CPT producing plants; G653 in all fungi except *N. aurantialba* which had A653 like human DNA Top I;
G717V/F	G717 in the active site helps in conformational flexibility that modulates CPT binding. Mutation of G717/V/F can confer CPT resistance	[13]	G717 found in all sequences, except in *N. aurantialba* which has a T717, and *O. pumila* which again has a S717
T729/A/K/E	T729 is located in the hydrophobic cavity and its integrity is necessary for facilitating the CPT-DNA binding and subsequent sensitivity to CPT. T729/A/K/E mutation causes CPT resistance.	[59]	T729 found in all sequences with a gap in one of the DNA Top I sequence from *Cladosporium cladosporioides MD2*
E356	Residue present in drug (CPT) binding site of DNA Top I	[13]	Highly conserved in all sequences involved. No mutation
H367	Residue present in drug (CPT) binding site of DNA Top I	[45]	Highly conserved in all sequences involved. No mutation
V502	Residue present in drug (CPT) binding site of DNA Top I	[13]	Highly conserved in all sequences involved. No mutation
Y619	Residue present in drug (CPT) binding site of DNA Top I	[13]	Highly conserved in all sequences involved. No mutation
D725	Residue present in drug (CPT) binding site of DNA Top I	[45]	Highly conserved in all sequences involved with a gap in one of the DNA Top I sequence from *Cladosporium cladosporioides MD2*. No mutation

References

58. Chang, J. Y., Liu, J. F., Juang, S. H., Liu, T. W. & Chen, L. T. Novel mutation of topoisomerase I in rendering cells resistant to camptothecin. *Cancer Res.* **62**, 3716–3721 (2002).
59. Losasso, C. *et al.* A single mutation in the 729 residue modulates human DNA topoisomerase IB DNA binding and drug resistance. *Nucleic Acids Res.* **36**, 5635–5644 (2008).
